# Standard School Books

**Published:** 1895-08

**Authors:** 


STANDARD SCHOOL BOOKS.

Messrs. Funk & Wagnalls, the publishers of the now celebrated *Standard Dictionary*, so frequently referred to in this journal, are about to publish a series of school books, modeled somewhat on the plan of the *Standard Dictionary*, and serving as complements to it. These books are as follows: *Students' Standard Dictionary*, *Students' Standard Synonyms*, *Students' Standard Speller*, *Standard First*, *Second*, *Third*, *Fourth*, and *Fifth Readers*. All these will be noticed in these pages as they appear.

